# Highly Discriminative Genotyping of *Mycobacterium abscessus* Complex Using a Set of Variable Number Tandem Repeats in China

**DOI:** 10.3389/fmicb.2021.802133

**Published:** 2022-01-31

**Authors:** Lihua Huang, Haoran Li, Weicong Ren, Xuxia Zhang, Yuanyuan Shang, Yi Liu, Aimei Liu, Yu Pang

**Affiliations:** ^1^Longtan Hospital of Guangxi Zhuang Autonomous Region, Liuzhou, China; ^2^Department of Bacteriology and Immunology, Beijing Chest Hospital, Beijing Tuberculosis and Thoracic Tumor Research Institute, Capital Medical University, Beijing, China

**Keywords:** *Mycobacterium abscessus* complex, subspecies, polymorphism, genotyping, MIRU-VNTR

## Abstract

In this study, our aims were to comparatively analyze the power of variable number tandem repeat (VNTR) typing to discriminate isolates within subspecies and to identify a potential genetic marker for better molecular typing of *Mycobacterium abscessus* complex (MABC) strains. A total of 103 clinical MABC isolates were collected from a nationwide cross-sectional study in China. Eighteen VNTR loci were chosen to genotype the MABC isolates. Of the 103 clinical MABC isolates, there were 76 (73.8%) *M. abscessus* subsp. *abscessus* (MAA) and 27 (26.2%) *M. abscessus* subsp. *massiliense* (MAM) isolates. Among the patients with MAA lung diseases, the percentage of patients older than 45 years (67.1%) was significantly higher than that of patients with MAM lung diseases [33.3%, adjusted odds ratio (aOR) = 0.36, 95% CI = 0.13–0.98, *p* = 0.046]. Fifteen VNTR loci were designated as being “highly discriminant” in our sample, except for TR109. The total of 103 MABC isolates were fully discriminated into 103 unique patterns by an 18-locus VNTR set [Hunter–Gaston Discriminatory Index (HGDI) = 1.000], of which the inclusion of the top 12 loci yielded a comparative HGDI value (HGDI = 0.9998). Remarkably, the order of the diversity of the VNTR loci showed significant difference between the MAA and MAM isolates. TR137 and TR2, two loci with high diversity indices for the MAA isolates, only yielded poor discriminatory power for the MAM isolates; the allelic diversity (*h*) values were 0.0000 and 0.2621, respectively. A detailed analysis of TR137 in combination with the other 17 VNTR loci showed that the combination of TR137–TR2 could fully distinguish MAA from MAM isolates. In conclusion, our data revealed that MAA is more prone to affect elderly patients. Additionally, the population structure of the MABC isolates circulating in China has high diversity. The combined use of the TR137 and TR2 loci provides a simple criterion for the precise identification of MABC to the subspecies level.

## Introduction

Worldwide, infections due to non-tuberculous mycobacteria (NTM) appear to be increasing (Thomson and NTM working group at Queensland TB Control Centre and Queensland Mycobacterial Reference Laboratory, 2010; Pang et al., 2017). Despite controversy as to the human-to-human transmission of NTM, their ubiquitous distribution in the environment increases the risk of NTM diseases in humans, especially in immunosuppressed and immunocompetent individuals (Bange et al., 2001; Ricketts et al., 2014; Harris et al., 2015; McShane and Glassroth, 2015). Approximately 200 NTM species have been identified, of which the *Mycobacterium avium* complex (MAC), *Mycobacterium kansasii*, and the *Mycobacterium abscessus* complex (MABC) are the major causative agents of human NTM diseases (Johansen et al., 2020). Significant differences in the geographic of NTM have been noted, reflecting diversities of their distribution in environmental niche across geographical locations (Pang et al., 2017).

MABC is composed of the most common rapid growing mycobacteria that cause human diseases, accounting for 23.1% of pulmonary infections due to NTM in China (Tan et al., 2021). It can also affect multiple organs and lead to severe respiratory, skin, and mucosal infections in humans (Ryan and Byrd, 2018; Kwak et al., 2019). More importantly, clinical treatment of MABC infections remains difficult, as it is inherently resistant to the majority of available antibiotics (Nessar et al., 2012; Nie et al., 2014; Lopeman et al., 2019). Patients infected with MABC are generally associated with poor clinical outcomes, particularly individuals with impaired immunity and underlying lung diseases. MABC comprises three closely related species: *M. abscessus* subsp. *abscessus* (MAA), *M. abscessus* subsp. *massiliense* (MAM), and *M. abscessus* subsp. *bolletii* (MAB) (Johansen et al., 2020). Despite sharing a high sequence similarity within MABC subspecies, a clinically significant difference has been observed between MAA and MAM, which was primarily caused by the clarithromycin (CLA) resistance-associated gene *erm*(41) (Kim et al., 2010; Brown-Elliott et al., 2015). MAM has a truncated *erm*(41) gene, whereas MAA has a functional *erm*(41) gene corresponding to inducible resistance, thereby leading to poorer outcomes (Choi et al., 2012). This sequence divergence indicates significant evolutionary differences between these two neighboring mycobacteria. Further comparative investigation of their genetic diversity will extend our knowledge of these microorganisms.

Variable number tandem repeat (VNTR) analysis has been widely accepted as an alternative to the differentiation of mycobacteria strains (Wong et al., 2012; Iakhiaeva et al., 2013; Zhao et al., 2014). For MABC, an 18-locus VNTR combination has been well established for genotyping strains belonging to this species and has shown promisingly good discriminatory power with a panel of MABC strains isolated from NTM pulmonary disease (NTM-PD) patients (Wong et al., 2012). In the study described here, we extended that initial study by applying VNTR typing to a large strain panel comprising MAA and MAM strains collected from a national surveillance in China. Our aims “were to comparatively analyze the power of VNTR typing to discriminate isolates within these subspecies and to identify a potential genetic marker for better molecular typing of MABC strains.

## Materials and Methods

### Bacterial Strains

A total of 103 clinical MABC isolates were collected from the China Non-tuberculous Mycobacteria Surveillance Study (CNTMS), a nationwide cross-sectional study in China consecutively enrolling smear-positive patients with clinical symptoms suggestive of tuberculosis from December 2019 to June 2020 (Tan et al., 2021). The demographic and clinical variables were obtained from the electronic patient records in each hospital.

All NTM isolates were transferred to the Beijing Chest Hospital, Capital Medical University, for further analysis. Prior to species identification, the isolates were recovered on Löwenstein–Jensen (L-J) medium for 7 days.

### Species Identification

Fresh colonies were harvested from the surface of the L-J medium with a plastic loop. A bacterial suspension of McFarland 1.0 standard was prepared for genomic DNA extraction using the cetyltrimethylammonium bromide (CTAB) NaCl method as previously described (Devonshire et al., 2015). Multilocus sequence analysis was used as the gold standard to differentiate the MABC isolates into the subspecies level. Primary identification to the species level was performed by ruling out membership of the NTM species using the MeltPro^®^ Mycobacteria Identification Kit (Zeesan Biotech, Xiamen, China) (Xu et al., 2019), followed by sequencing of the entire *rpoB* and *hsp65* genes for the differentiation of the MABC isolates. The following primer pairs were used for PCR amplification as previously reported: *rpoB* forward: 5′-CTA GCG GTA GTC GCT GTA GC-3′, *rpoB* reverse: 5′-GTG CTC GAC GTC AAC TTC TT-3′; *hsp65* forward; 5′-ATG GCC AAG ACA ATT GCG TA-3′, *hsp65* reverse: 5′-TTA GAA GTC CAT GCC ACC CA-3′. After purification with the QIAEXII Gel Extraction Kit (Qiagen, Hilden, Germany), the amplicons were sent to the Tsingke Company (Beijing, China) for sequencing service. DNA sequences were blasted with the homologous sequences of the reference mycobacterial strains using the Basic Local Alignment Search Tool (BLAST) from NCBI^1^. The DNA sequences of *rpoB* and *hsp65* in this study have been submitted to GenBank.

### Genotyping

Eighteen VNTR loci were chosen to genotype the MABC isolates in this study (Wong et al., 2012). The primers for VNTR loci were synthesized by the Tsingke Company (Beijing, China). The PCR reaction mixture was prepared as follows: 10 μl 2 × Taq PCR mixture (TIANGEN, Beijing, China), 0.5 μM each primer, and 2.5 μl genomic DNA. The final volume was adjusted to 20 μl with distilled water, and the reaction mixture was then amplified with initial denaturation at 94°C for 5 min, followed by 35 amplification cycles of denaturation at 94°C for 30 s, annealing at 62°C for 30 s, and extension at 72°C for 30 s, and finally extended at 72°C for 7 min. The PCR products were electrophoresed in 2% agarose gel stained with 4S Green Plus (TaKaRa, Shiga, Japan). The reference MAA strain (ATCC 19977) was run as an additional control of accuracy and for the accurate interpretation of the repeat number of each VNTR locus. The repeat numbers of different alleles were calculated according to the allele size range and the basic unit length.

### Statistical Analysis

The discrimination ability of each tandem repeat (TR) was calculated and analyzed using Hunter–Gaston Discriminatory Index (HGDI) scores according to the method by Hunter and Gaston (1988). Data of the 18 TR loci of the MABC isolates were analyzed using the BioNumerics software (version 5.0; Applied Maths, Sint-Martens-Latem, Belgium). A dendrogram was generated in BioNumerics using the average linkage clustering method (unweighted pair group method with arithmetic mean, UPGMA). One-way ANOVA was used to compare the differences between multiple groups. The difference was considered statistically significant when *p* < 0.05. All calculations were performed using SPSS 22.0 software for Windows (SPSS Inc., Chicago, IL, United States).

## Results

### Demographic and Clinical Characteristics of Patients

Among the 103 clinical MABC isolates circulating in China, 76 (73.8%) were MAA and 27 (26.2%) were MAM isolates. The baseline demographic and clinical characteristics are shown in Table 1. Of the patients, 53.4% (55/103) were females, and patients aged > 45 years (60/103) accounted for the majority of the studied participants. In addition, 87 cases (84.5%) had previous tuberculosis (TB) episodes. We further compared the percentages of the demographic and clinical characteristics of patients with MAA and MAM lung diseases. Among the patients with MAA lung diseases, the percentage of patients older than 45 years (67.1%) was significantly higher than that of patients with MAM lung diseases [33.3%, adjusted odds ratio (aOR) = 0.36 95% CI = 0.13–0.98, *p* = 0.046]. In contrast, multiple logistic regression analysis revealed no statistically significant differences in several other demographic and clinical characteristics (*p* > 0.05).

**TABLE 1:** Differences in the characteristics between *Mycobacterium abscessus* subsp. *abscessus* (MAA) and *M. abscessus* subsp. *massiliense* (MAM).

Characteristics	Total, *n* = 103 (%)	MAA, *n* = 76 (%)	MAM, *n* = 27 (%)	OR (95% CI)	*p*-value	Adjusted OR (95% CI)	*p*-value
**Sex**							
Male	48 (46.6)	40 (52.6)	8 (29.6)	1	References	1	References
Female	55 (53.4)	36 (47.4)	19 (70.4)	2.64 (1.03–6.76)	0.04	1.86 (0.68–5.13)	0.229
Age (years)							
16–44	43 (41.7)	25 (32.9)	18 (66.7)	1	References	1	References
≥ 45	60 (58.3)	51 (67.1)	9 (33.3)	0.25 (0.10–0.62)	0.002	0.36 (0.13–0.98)	0.046
**Ethnicity**							
Han	98 (95.1)	73 (96.1)	25 (92.6)	1	References	–	–
Other	5 (4.9)	3 (3.9)	2 (7.4)	1.95 (0.31–12.33)	0.604	–	–
**Previous TB episode**							
Yes	87 (84.5)	64 (84.2)	23 (85.2)	1	References	–	–
No	16 (15.5)	12 (15.8)	4 (14.8)	0.93 (0.27–3.17)	1	–	–
**Comorbidity**							
Yes	39 (37.9)	34 (44.7)	5 (18.5)	1	References	1	References
No	64 (62.1)	42 (55.3)	22 (81.5)	3.56 (1.22–10.40)	0.016	0.40 (0.13–1.24)	0.112
**Symptoms**							
Cough	80 (77.7)	57 (75.0)	23 (85.2)	1.92 (0.59–6.25)	0.42	–	–
Fever	16 (15.5)	14 (18.4)	2 (7.4)	0.35 (0.08–1.67)	0.227	–	–
Hemoptysis	14 (13.6)	12 (15.8)	2 (7.4)	0.43 (0.65–1.08)	0.346	–	–

*TB, tuberculosis.*

### Allelic Diversity of the MABC Isolates and Distribution of Variable Number Tandem Repeat Allele Numbers

**FIGURE 1:**
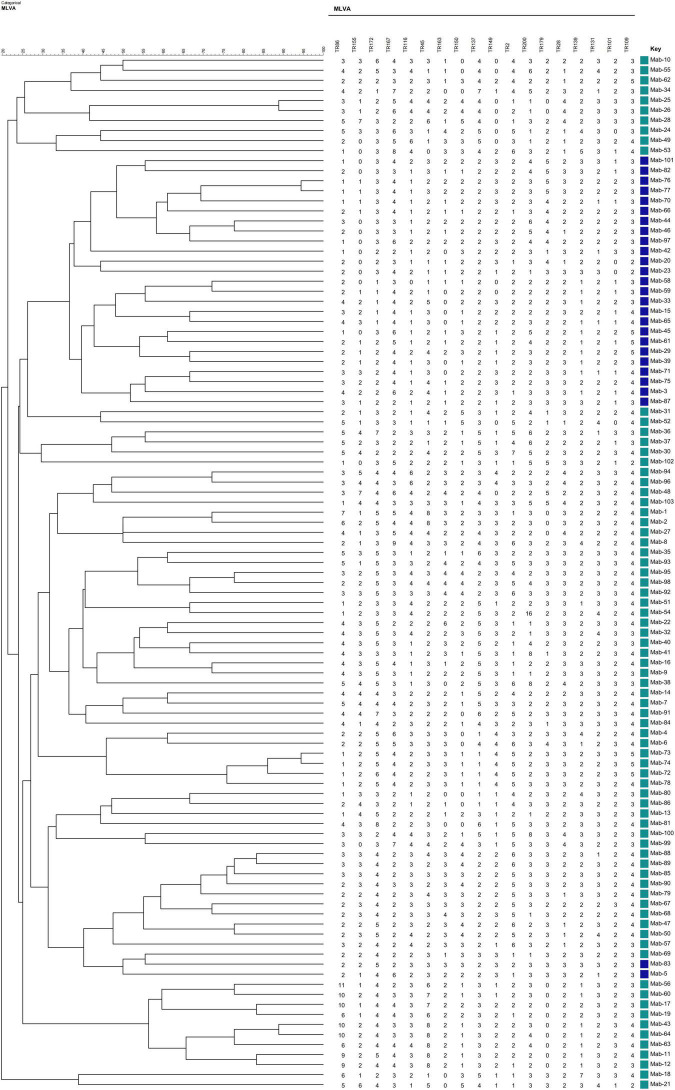
Phylogenetic tree of 103 *Mycobacterium abscessus* complex (MABC) strains based on variable number tandem repeat (VNTR) typing created using the BioNumerics v5.0 (Applied Math) the unweighted pair group method with arithmetic mean (UPGMA).

Allelic diversity (*h*) is a good index of the discriminatory power provided by each VNTR locus. On the basis of this index, 15 VNTR loci were designated as being “highly discriminant” (*h* > 0.6) in our sample, except for TR109, which was designated as moderately discriminant (0.3 ≤ *h* ≤ 0.6; Table 2). The total of 103 MABC isolates were fully discriminated into 103 unique patterns by the 18-locus VNTR set (HGDI = 1.000; Figure 1), of which the inclusion of the top 12 loci yielded a comparative HGDI value (HGDI = 0.9998).

**TABLE 2:** Combined HGDI for 103 independent *Mycobacterium abscessus* complex (MABC) isolates.

Locus	No. of isolates with VNTR repeat units													HGDI^a^	Combined HGDI^b^					
	0	1	2	3	4	5	6	7	8	9	10	11	16		TR86 to TR172	TR86 to TR116	TR86 to TR200	TR86 to TR131	TR86 to TR101	All loci
TR86		18	27	19	16	11	4	1	0	2	4	1		0.8357	0.9882	0.9967	0.9998	1.0000	1.0000	1.0000
TR155	14	23	30	22	10	1	1	2						0.7990						
TR172		6	15	27	26	24	2	2	1					0.7955						
TR167			25	30	29	7	8	2	1	1				0.7733						
TR116	1	28	26	26	19	0	3							0.7712						
TR45	1	10	36	30	12	2	3	2	7					0.7712						
TR163	13	22	34	26	7	0	1							0.7685						
TR150	9	27	37	15	11	4								0.7679						
TR137		7	40	19	15	18	3	1						0.7653						
TR149	11	23	27	34	8									0.7624						
TR2		18	42	5	9	19	9	1						0.7588						
TR200		11	29	40	9	6	4	3					1	0.7523						
TR179	14	5	33	37	7	7								0.7455						
TR28		12	37	44	10									0.6720						
TR139		19	57	21	4	1	0	1						0.6225						
TR131		8	41	48	6									0.6210						
TR101	4	15	58	26										0.6023						
TR109			4	42	50	7								0.5978						

*^a^HGDI, Hunter–Gaston Discriminatory Index for each variable number tandem repeat (VNTR) locus.*

*^b^The shaded portions show the combined HGDI values for TR86–TR172, TR86–TR116, TR86–TR200, TR86–TR131, and TR86–TR101, showing increasing discriminatory power from 0.9882 for 3 loci combined to a maximum of 1.0000 for 16 loci combined (matching the overall HGDI of 0.9563 for all 18 tandem repeats (TRs) combined, as shown).*

### Subspecies Diversity of the Variable Number Tandem Repeat Loci

**FIGURE 2:**
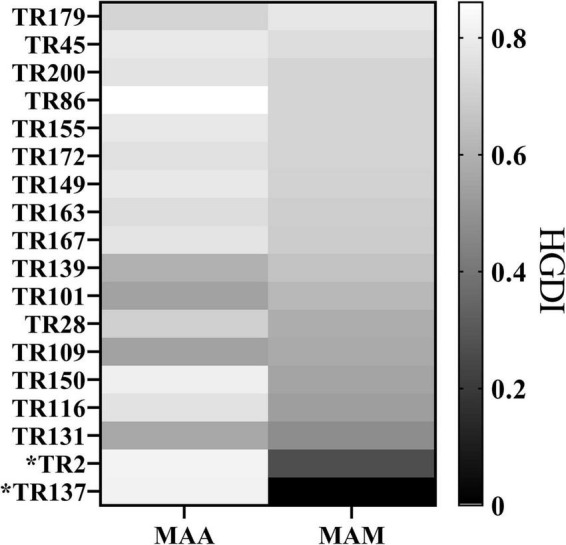
Hunter–Gaston Discriminatory Index (HGDI) for each variable number tandem repeat (VNTR) locus stratified to different subspecies showing that the TR137 and TR2 loci exhibited the potential to distinguish subspecies within *Mycobacterium abscessus* complex (MABC).

We further analyzed the discriminatory power of each VNTR locus stratified to different subspecies (Figure 2 and Supplementary Tables 1, 2). For the MAA isolates, the majority of the VNTR loci showed high–moderate diversity indices, consisting of TR86, TR2, and TR137. Four VNTR loci (TR101, TR109, TR131, and TR139) achieved a moderate diversity index. For the MAM isolates, high and moderate diversity indices were recorded in 11 and 5 VNTR loci, respectively. Remarkably, the order of the diversity of the VNTR loci showed significant difference between the MAA and MAM isolates. TR137 and TR2, two loci with high diversity indices for the MAA isolates, only yielded poor discriminatory power for the MAM isolates; the *h* values were 0.0000 and 0.2621, respectively. The repeat number of the MAM isolates at both TR2 and TR137 loci was less than 3. Notably, all MAM isolates had two repeats at the TR137 locus, whereas the repeat number of the MAA isolates at this locus ranged from 1 to 7, indicating that TR137 exhibited subspecies specificity within MABC.

### Accuracy of TR137 Plus TR2 for Species Identification Among MABC Isolates

**FIGURE 3:**
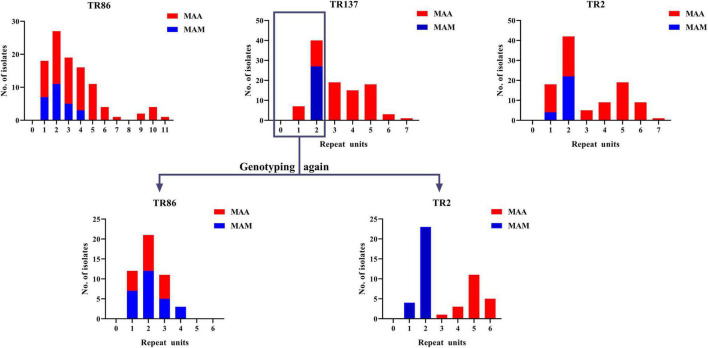
Statistical analysis of *Mycobacterium abscessus* subsp. *abscessus* (MAA) and *M. abscessus* subsp. *massiliense* (MAM) repeats units at 18 tandem repeat (TR) loci showing that the repeat units of the MAM isolates were less than 3 only at TR137 and TR2 of the 18 TR loci (top). When the strains with TR137 repeat units less than 3 were detected again, only the TR2 locus could completely distinguish MAM from MAA (bottom).

**FIGURE 4:**
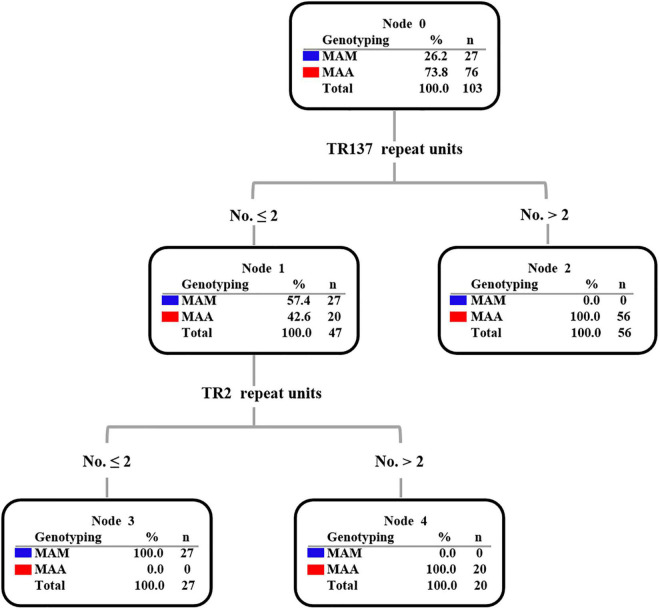
Decision tree diagram based on tandem variable number tandem repeat (VNTR) showing that TR137 combined with the TR2 locus can distinguish *Mycobacterium abscessus* subsp. *abscessus* (MAA) and *M. abscessus* subsp. *massiliense* (MAM) in the *Mycobacterium abscessus* complex (MABC).

Subsequently, the potential VNTR locus combination that could be used for species identification was investigated. A detailed analysis of TR137 combined with the other 17 VNTR loci showed that the TR137–TR2 combination could fully distinguish MAA from MAM isolates (Figure 3). As shown in Figure 4, the majority of MAA with a TR137 repeat unit higher than 2 could be identified by TR137 screening. Thereafter, the remaining MABC isolates with a TR137 repeat unit equal to or lower than 2 were completely distinguished into MAA (TR2 repeat units > 2) and MAM (TR2 repeat units ≤ 2) by TR2 screening.

## Discussion

MABC has attracted increasing attention due to being the most chemotherapy-resistant member of NTM (Johansen et al., 2020). As the second prevalent NTM species in China, MABC accounts for 23.1% of patients with NTM-PD, of which 56.9% were MAA and the other 43.1% were MAM (Pang et al., 2017; Tan et al., 2021). We firstly identified the difference in age distribution between MAA and MAM. Compared with individuals infected with MAM, those with MAA were more frequently > 45 years old. Considering that human immunity is known to significantly decline with increasing age (Valiathan et al., 2016), MAA seems to be less virulent than MAM. In line with our hypothesis, previous clinical data found that only one-third of MAA pulmonary infection presented obvious symptoms, whereas all patients with MAM pulmonary infection had obvious symptoms (Shin et al., 2013), indicating possible differences in the virulence between these neighboring subspecies. However, the reliability of the hypothesis is limited by the small cohort size included in the present study. An experimental investigation is needed to determine whether any differences in host responses and clinical outcomes exist among animals infected with these subspecies.

Our genotyping data with the classical 18-locus VNTR set revealed that the population structure of the MABC isolates circulating in China has high diversity. On one hand, we evaluated the optimal VNTR locus combination for genotyping MABC isolates. A comprehensive analysis of the genotyping data revealed that the inclusion of the top 12 loci yielded a comparative HGDI value (HGDI = 0.9998) relative to that of the 18-locus set (HGDI = 1.0000). Considering labor and cost, this 12-locus set may be a cost-effective tool for differentiating the MABC isolates in China. On the other hand, the high diversity of the MAC isolates may be attributed to their widespread geographic distribution across China, which were lacking of epidemiological links. Besides, our preliminary data support that there was no person-to-person transmission among the general population, despite potential nosocomial transmission of MABC occurring in cystic fibrosis patients in the United Kingdom (Bryant et al., 2013). The low risk of transmission of MABC in the community reflects the attenuated virulence of this NTM species. In addition, NTM species are well equipped to survive in the environment. Notably, MAC could survive in amoeba trophozoites and subsequent cyst stages (Drancourt, 2014). Its remarkable survival in amoeba would provide benefits for the transition of MABC from environmental organisms to human pathogens, thereby accelerating the accumulation of genetic diversity. Taken together, our data demonstrate that the MABC isolates causing pulmonary diseases primarily originate from the environmental niche. Further comparative investigation on environmental MABC isolates will extend our knowledge on the evolutionary pathway of MABC through the acquisition of important virulence genes.

Another interesting finding in our study was that the repeat numbers of TR137 exhibited significant species diversity between MAA and MAM. TR137 was present as one to seven copies in MAA, while MAM showed one pattern with two copies of TR137. Upon combing the genotyping results of TR2, another VNTR locus with discriminatory power for resolving subspecies, complete identity agreement was found between this novel assay and the reference method. Similar to our observation, Choi and colleagues found that two TR loci could be used to discriminate MAA and MAM *via* different copy numbers of VNTR11 and VNTR23 (Choi et al., 2011). In addition, our results suggest that genotypes in particular VNTR targets represent an evolution marker of the microdiversity of MABC, thus being involved in the pathogenesis of MAA and MAM lung diseases (Shin et al., 2013). The combined use of the TR137 and TR2 loci provides a simple criterion for the precise identification of MABC to the subspecies level. Compared with the conventional Sanger sequencing or other molecular methods, it only requires standard PCR and gel electrophoresis facilities rather than expensive equipment. Besides, with a 4-h procedure time and a reagent cost of United States $3, this method seems affordable and could be easily available in routine microbiology laboratories, especially laboratories in resource-limited settings.

We also acknowledge several obvious limitations of the present study. Firstly, despite the enrollment of all MABC isolates collected in the national surveillance, the small sample size may limit the confidence of our conclusion. Further validation of this novel two-locus VTNR set in a larger series of MABC patients is required to validate our findings. Secondly, another important subspecies, *M. abscessus* subsp. *bolletii* (MAB), was not identified in the NTM isolates from China, which was inconsistent with our previous observations (Pang et al., 2017). As a consequence, our observation should be confirmed in clinical MAB isolates. Thirdly, clarithromycin susceptibility was not determined in this study, which hampered further analysis of the association between the VNTR pattern and the minimal inhibitory concentration (MIC) of clarithromycin. Lastly, a previous report revealed that the MABC genotype influenced mycobacterial virulence (Shin et al., 2013), thus presenting a remarkable difference in clinical disease phenotype and disease progression. Unfortunately, the clinical outcomes of the patients infected with MABC were not collected in this observational study. Further study is urgently required for elucidating the correlation between phenotype and genotype among the MABC isolates.

## Conclusion

Our data revealed the difference in age distribution between MAA and MAM, and MAA was more prone to affect elderly patients. In addition, the population structure of the MABC isolates circulating in China showed a high diversity, indicating that the MABC isolates causing pulmonary diseases primarily originate from the environmental niche. The combined use of the TR137 and TR2 loci provided a simple criterion for the precise identification of MABC to the subspecies level. To extend these results, studies of the association between the clinical outcomes, distinct pathogenesis, and VNTR profiles are warranted.

## Data Availability Statement

The datasets presented in this study can be found in online repositories. The names of the repository/repositories and accession number(s) can be found below: GenBank, OL704521 - OL704623, OL704624 - OL704726.

## Ethics Statement

This study was approved by the Ethics Committee of Beijing Chest Hospital, Capital Medical University. Informed consent from all participating subjects was obtained.

## Author Contributions

YP, WR, and AL conceptualized the study. YP and YL designed the methodology. LH, YS, and XZ performed formal analysis. LH, AL, and XZ conducted the investigation. HL, WR, and YS curated the data. HL, YP, and LH prepared the original draft. YP, AL, and HL reviewed and edited the manuscript. YL, YP, and AL acquired funding. All authors contributed to the manuscript and approved the submitted version.

## Conflict of Interest

The authors declare that the research was conducted in the absence of any commercial or financial relationships that could be construed as a potential conflict of interest.

## Publisher’s Note

All claims expressed in this article are solely those of the authors and do not necessarily represent those of their affiliated organizations, or those of the publisher, the editors and the reviewers. Any product that may be evaluated in this article, or claim that may be made by its manufacturer, is not guaranteed or endorsed by the publisher.

## Footnotes

1
http://www.ncbi.nlm.nih.gov/BLAST
