# Supplementary material for: Highly Discriminative Genotyping of Mycobacterium abscessus Complex Using a Set of Variable Number Tandem Repeats in China
Source: Front Microbiol. 2022 Jan 31;12:802133. doi: 10.3389/fmicb.2021.802133 (PMC8841818; doi:10.3389/fmicb.2021.802133)
Supplement: Supplementary file 1 [file Data_Sheet_1.docx]

| **Supplementary Table 1** Combined HGDI for 76 independent MAA isolates | | | | | | | | | | | | | | | | | | | | |
| --- | --- | --- | --- | --- | --- | --- | --- | --- | --- | --- | --- | --- | --- | --- | --- | --- | --- | --- | --- | --- |
|  | No. of isolates with VNTR repeat units no. | | | | | | | | | | | | |  | Combined HGDIb | | | | | |
|  |  |  |  |  |  |  |  |  |  |  |  |  |  |  | TR86 to | TR86 to | TR86 to | TR86 to | TR86 to |  |
| Locus | 0 | 1 | 2 | 3 | 4 | 5 | 6 | 7 | 8 | 9 | 10 | 11 | 16 | HGDI^a^ | TR137 | TR150 | TR45 | TR131 | TR101 | All loci |
| TR86 |  | 11 | 15 | 14 | 13 | 11 | 4 | 1 | 0 | 2 | 4 | 1 |  | 0.8607 | 0.9821 | 0.9905 | 0.9965 | 1.0000 | 1.0000 | 1.0000 |
| TR2 |  | 14 | 19 | 5 | 9 | 19 | 9 | 1 |  |  |  |  |  | 0.8193 |  |  |  |  |  |  |
| TR137 |  | 7 | 13 | 19 | 15 | 18 | 3 | 1 |  |  |  |  |  | 0.8137 |  |  |  |  |  |  |
| TR150 | 9 | 21 | 20 | 11 | 11 | 4 |  |  |  |  |  |  |  | 0.8063 |  |  |  |  |  |  |
| TR45 | 1 | 6 | 26 | 21 | 9 | 1 | 3 | 2 | 7 |  |  |  |  | 0.7856 |  |  |  |  |  |  |
| TR155 | 4 | 13 | 25 | 20 | 10 | 1 | 1 | 2 |  |  |  |  |  | 0.7825 |  |  |  |  |  |  |
| TR149 | 9 | 17 | 20 | 22 | 8 |  |  |  |  |  |  |  |  | 0.7821 |  |  |  |  |  |  |
| TR167 |  |  | 22 | 25 | 15 | 6 | 4 | 2 | 1 | 1 |  |  |  | 0.7691 |  |  |  |  |  |  |
| TR200 |  | 10 | 23 | 27 | 5 | 4 | 3 | 0 | 3 |  |  |  | 1 | 0.7646 |  |  |  |  |  |  |
| TR116 |  | 11 | 18 | 25 | 19 | 0 | 3 |  |  |  |  |  |  | 0.7607 |  |  |  |  |  |  |
| TR172 |  | 1 | 6 | 16 | 25 | 23 | 2 | 2 | 1 |  |  |  |  | 0.7579 |  |  |  |  |  |  |
| TR163 | 6 | 10 | 27 | 25 | 7 | 0 | 1 |  |  |  |  |  |  | 0.7432 |  |  |  |  |  |  |
| TR179 | 14 | 4 | 24 | 30 | 1 | 3 |  |  |  |  |  |  |  | 0.7154 |  |  |  |  |  |  |
| TR28 |  | 10 | 26 | 30 | 10 |  |  |  |  |  |  |  |  | 0.7018 |  |  |  |  |  |  |
| TR139 |  | 9 | 45 | 16 | 4 | 1 | 0 | 1 |  |  |  |  |  | 0.5958 |  |  |  |  |  |  |
| TR131 |  | 3 | 22 | 45 | 6 |  |  |  |  |  |  |  |  | 0.5653 |  |  |  |  |  |  |
| TR101 | 2 | 4 | 45 | 25 |  |  |  |  |  |  |  |  |  | 0.5449 |  |  |  |  |  |  |
| TR109 |  |  | 2 | 25 | 45 | 4 |  |  |  |  |  |  |  | 0.5449 |  |  |  |  |  |  |
| ^a^HGDI, Hunter-Gaston discriminatory index for each VNTR locus. | | | | | | | | | | | | | | | | | | | | |
| ^b^The shaded portions show the combined HGDIs for TR86 to TR137, TR86 to TR150, TR86 to TR45, TR86 to TR131, and TR86 to TR101, showing increasing discriminatory power from 0.9821 for 3 loci combined to a maximum of 1.0000 for 16 loci combined (matching the overall HGDI of 0.9563 for all 18 TRs combined, as shown). | | | | | | | | | | | | | | | | | | | | |

| **Supplementary Table 2** Combined HGDI for 27 independent MAM isolates | | | | | | | | | | | | | | | | | | | | |
| --- | --- | --- | --- | --- | --- | --- | --- | --- | --- | --- | --- | --- | --- | --- | --- | --- | --- | --- | --- | --- |
|  | No. of isolates with VNTR repeat units no.: | | | | | | | | | | | | |  | Combined HGDI^b^ | | | | | |
|  |  |  |  |  |  |  |  |  |  |  |  |  |  |  | TR179 to | TR179 to | TR179 to | TR179 to | TR179 to |  |
| Locus | 0 | 1 | 2 | 3 | 4 | 5 | 6 | 7 | 8 | 9 | 10 | 11 | 16 | HGDI^a^ | TR86 | TR155 | TR172 | TR149 | TR101 | All loci |
| TR179 |  | 1 | 9 | 7 | 6 | 4 |  |  |  |  |  |  |  | 0.7778 | 0.9858 | 1.0000 | 1.0000 | 1.0000 | 1.0000 | 1.0000 |
| TR45 |  | 4 | 10 | 9 | 3 | 1 |  |  |  |  |  |  |  | 0.7436 |  |  |  |  |  |  |
| TR86 |  | 7 | 12 | 5 | 3 |  |  |  |  |  |  |  |  | 0.7151 |  |  |  |  |  |  |
| TR200 |  | 1 | 6 | 13 | 4 | 2 | 1 |  |  |  |  |  |  | 0.7151 |  |  |  |  |  |  |
| TR155 | 10 | 10 | 5 | 2 |  |  |  |  |  |  |  |  |  | 0.7123 |  |  |  |  |  |  |
| TR172 |  | 5 | 9 | 11 | 1 | 1 |  |  |  |  |  |  |  | 0.7123 |  |  |  |  |  |  |
| TR149 | 2 | 6 | 7 | 12 |  |  |  |  |  |  |  |  |  | 0.7066 |  |  |  |  |  |  |
| TR163 | 7 | 12 | 7 | 1 |  |  |  |  |  |  |  |  |  | 0.6923 |  |  |  |  |  |  |
| TR167 |  |  | 3 | 5 | 14 | 1 | 4 |  |  |  |  |  |  | 0.6866 |  |  |  |  |  |  |
| TR139 |  | 10 | 12 | 5 |  |  |  |  |  |  |  |  |  | 0.6553 |  |  |  |  |  |  |
| TR101 | 2 | 11 | 13 | 1 |  |  |  |  |  |  |  |  |  | 0.6182 |  |  |  |  |  |  |
| TR28 |  | 2 | 11 | 14 |  |  |  |  |  |  |  |  |  | 0.5812 |  |  |  |  |  |  |
| TR109 |  |  | 2 | 17 | 5 | 3 |  |  |  |  |  |  |  | 0.5726 |  |  |  |  |  |  |
| TR150 |  | 6 | 17 | 4 |  |  |  |  |  |  |  |  |  | 0.5527 |  |  |  |  |  |  |
| TR116 | 1 | 17 | 8 | 1 |  |  |  |  |  |  |  |  |  | 0.5328 |  |  |  |  |  |  |
| TR131 |  | 5 | 19 | 3 |  |  |  |  |  |  |  |  |  | 0.4758 |  |  |  |  |  |  |
| TR2 |  | 4 | 23 |  |  |  |  |  |  |  |  |  |  | 0.2621 |  |  |  |  |  |  |
| TR137 |  |  | 27 |  |  |  |  |  |  |  |  |  |  | 0.0000 |  |  |  |  |  |  |
| ^a^HGDI, Hunter-Gaston discriminatory index for each VNTR locus. | | | | | | | | | | | | | | | | | | | | |
| ^b^The shaded portions show the combined HGDIs for TR179 to TR86, TR179 to TR155, TR179 to TR172, TR179 to TR149, and TR179 to TR101, showing increasing discriminatory power from 0.9858 for 3 loci combined to a maximum of 1.0000 for 5 loci combined (matching the overall HGDI of 0.9563 for all 18 TRs combined, as shown). | | | | | | | | | | | | | | | | | | | | |
